# Accelerated *BRAF* mutation analysis using a fully automated PCR platform improves the management of patients with metastatic melanoma

**DOI:** 10.18632/oncotarget.25957

**Published:** 2018-08-14

**Authors:** Delphine Serre, Julia Salleron, Marie Husson, Agnès Leroux, Pauline Gilson, Jean-Louis Merlin, Lionnel Geoffrois, Alexandre Harlé

**Affiliations:** ^1^ Institut de Cancérologie de Lorraine, Département d’Oncologie Médicale, Vandoeuvre-lès-Nancy Cedex 54519, France; ^2^ Institut de Cancérologie de Lorraine, Cellule Data Biostatistiques, Vandoeuvre-lès-Nancy Cedex 54519, France; ^3^ Institut de Cancérologie de Lorraine, Biopathologie, Vandoeuvre-lès-Nancy Cedex 54519, France; ^4^ Université de Lorraine, CNRS UMR 7039 CRAN, Institut de Cancérologie de Lorraine, Service de Biopathologie, Vandoeuvre-lès-Nancy Cedex 54519, France

**Keywords:** metastatic melanoma, BRAF, automated real-time PCR, therapeutic management

## Abstract

**Background:**

Determination of BRAF status is important for the therapeutic management of patients with metastatic melanoma.

**Objectives:**

We evaluated the impact of a faster determination of BRAF mutational status on the delay between initial consultation and initiation of treatment.

**Results:**

For the FA-PCR group a median delay of 16 days [11;18] was observed between initial consultation and the implementation of treatment, which was significantly lower than that observed for the SOP group (26 days [20;46], *p* = 0.035).

**Conclusions:**

In comparison to using conventional SOP, using an FA-PCR platform for BRAF mutation analysis of patients with metastatic melanoma significantly reduced the delay in initiation of personalized therapy by 10 days.

**Materials and Methods:**

Analysis of the BRAF mutation status of eight formalin-fixed paraffin-embedded (FFPE) tissue samples was performed using a CE-IVD fully-automated (FA) PCR-based platform. The delay between initial consultation and the implementation of treatment was compared between these samples (FA-PCR group) and a retrospective group of 29 FFPE samples analysed by standard operating procedures (SOP group) using conventional PCR.

## INTRODUCTION

Mutation of the *BRAF* gene can be found in approximately 50% of advanced (stage IIIC) or metastatic (stage IV) melanomas [1].

It has been shown that targeted therapies blocking the MAP kinase pathway (combination of BRAF inhibitors *i.e.* vemurafenib and dabrafenib and MEK inhibitors *i.e.* trametinib, cobimetinib), improve the response rate and are of clinical benefit for 50% of patients with *BRAF*-mutated metastatic melanoma after six weeks of treatment [2, 3]. These therapies also significantly increase median overall survival from 6 to 22 months [4–7]. For patients with *BRAF* wild-type melanoma or those patients with *BRAF* mutations showing tumour progression after targeted therapy, immunotherapy (*i.e.* ipilimumab, nivolumab or pembrolizumab) is the standard treatment prior to chemotherapy. Clinical symptoms of the disease are common, and timely implementation of treatment could improve quality of life [8]. It is particularly important to determine the *BRAF* status of patients with a poor performance status or an aggressive disease, so that they can commence the correct treatment at the correct time.

Mutation of the *BRAF* gene is commonly assessed by conventional molecular biology assays such as PCR or immunohistochemistry (IHC), using formalin-fixed paraffin-embedded (FFPE) tissue samples [9]. Accelerated *BRAF* mutation analysis is achievable using a CE marked *in vitro* diagnostic (CE-IVD) fully-automated (FA) PCR-based platform. This platform allows the determination of *BRAF* mutational status in less than two hours, including sample preparation, and has been proven suitable for routine molecular diagnosis of patients with metastatic melanoma [10].

In this study, we used an FA-PCR platform to rapidly screen patients with advanced or metastatic melanoma for *BRAF* gene mutations. We then evaluated the impact of an accelerated determination of *BRAF* status on the delay between initial consultation and initiation of treatment.

## RESULTS

Thirty-nine patients were initially included in this trial. In the FA-PCR group, 10 patients were prospectively included between December 2015 and March 2016; two patients were excluded due to violation of inclusion criteria (one with a mucosal melanoma and one whose melanoma was at a localized stage). In the SOP group 29 consecutive patients were retrospectively included between January 2013 and November 2015. In total, 37 patients were finally included in this study (29 patients with retrospective inclusion [SOP group] and 8 patients with prospective inclusion [FA-PCR group]).

The population characteristics at initial diagnosis are presented in Table 1.

**Table 1:** Population characteristics at initial diagnosis

	SOP group *n* = 29	FA-PCR group *n* = 8
Primary cutaneous melanoma subtype	55.17% (15)	75% (6)
Superficial spreading nodular melanoma	6.9% (2)	12.5% (1)
Acral lentiginous melanoma	6.9% (2)	0% (0)
Non-classifiable	10.34% (3)	12.5% (1)
Unknown primary melanoma	20.69% (6)	0% (0)
	1 n/a	
Breslow’s depth (mm)	2 [0.81;2.7]	1.75 [0.95;3.5]
Regressive melanoma	12.50% (1)	50% (2)
	21 n/a	4 n/a
Mitotic index		
<1/mm^2^	16.67% (2)	0% (0)
1/mm^2^	83.33% (10)	100% (6)
	17 n/a	2 n/a
Ulceration	47.06% (8)	50% (3)
	12 n/a	2 n/a
Sentinel node resection	31.03% (9)	50% (4)
Invaded nodes in case of resection	77.78% (7)	75% (3)
Lymph node dissection	55.17% (16)	37.5% (3)
Number of nodes collected	12 [7;18]	24 [18;30]
Number of invasive nodes	1 [0;3]	0 [0;0]
AJCC Stage		
IA	0	16.67% (1)
IB	7.41% (2)	0% (0)
IIA	14.81% (4)	50% (3)
IIB	3.70% (1)	16.67% (1)
IIIA	14.81% (4)	16.67% (1)
IIIB	18.52% (5)	0% (0)
IIIC	3.70% (1)	0% (0)
IV	37.04% (10)	0% (0)
	2 n/a	2 n/a
Previous treatment	51.72% (15)	87.5% (7)
Chemotherapy or systemic treatment	26.67% (4)	14.29% (1)
Surgery	73.3% (11)	85.71% (6)

Results are expressed as percentage and frequency %(n) or as median and [inter-quartile range] - n/a: not available.

At initial consultation, *BRAF* mutational status was unknown for all of the patients in the FA-PCR group and for 11/29 (38%) patients in the SOP group.

The delay between reception of the FFPE samples and receipt of the results by the oncologist was reduced using FA-PCR (0 [0;1] versus 7 days [7;12]) *p* < 0.001, Table 2).

**Table 2:** Comparison of the time delays (days) between the FA-PCR group (*n* = 8) and the SOP group with unknown *BRAF* mutational status (*n* = 11)

	FA-PCR group	SOP group with unknown *BRAF* mutational status	*p*-value^*^
Delay between oncologist consultation and prescription to determine BRAF status	0 [0;2] (0–6)	0 [0;14] (0–33)	0.316
Delay between prescription to determine BRAF status and request for FFPE sample	0 [0;0] (0–3)	0 [0;10] (0–16)	0.133
Delay between request for FFPE sample and reception of FFPE sample	6 [0;7] (0–9)	3 [0;8] (0–14)	0.798
Delay between reception of FFPE sample and reception of the results by the oncologist	0 [0;1] (0–5)	7 [7;12] (3–21)	<0.001
Delay between reception of the results by the oncologist and implementation of treatment	7 [6;13] (3–32)	4 [1;10] (0–28)	0.319
Delay between initial oncologist consultation and implementation of personalized treatment	16 [11;18] (6–44)	26 [20;46] (14–64)	0.035

Results are expressed as median, [inter-quartile range] and (range).

^*^ Mann–Whitney *U* test.

For the FA-PCR group, the median time taken between initial consultation and the implementation of treatment was 16 days [11;18]. A median of 6 days [0;7] was observed between prescription for analysis of *BRAF* gene mutational status and reception of the sample by the laboratory. For 7 of the patients (87.5%) the results were reported to the oncologist within the same day or the day following the analyses. The median duration from receipt of the results by the oncologist to implementation of the treatment was 7 days [6;13] (Table 2).

**Figure 1:**
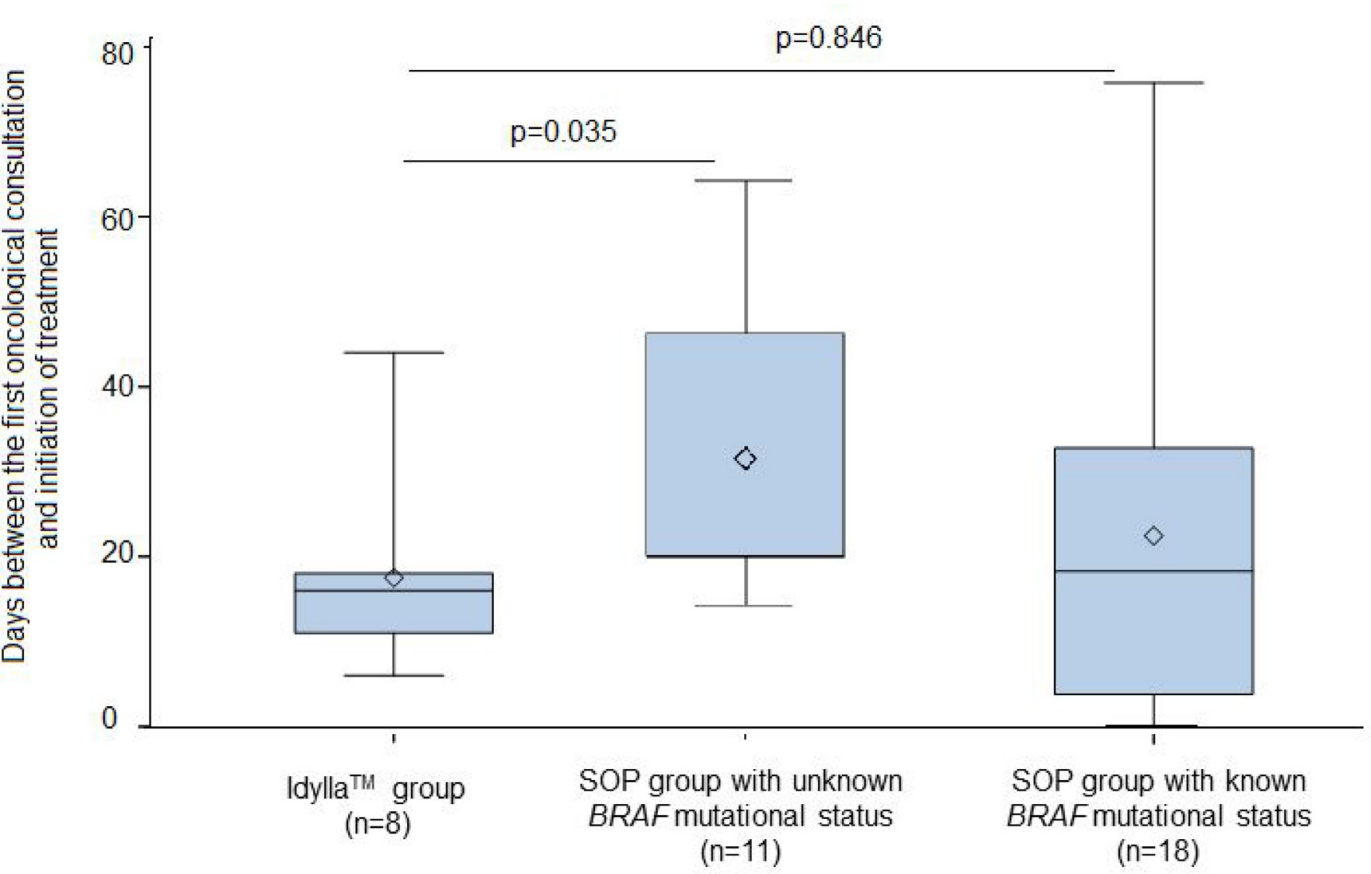
Comparison of the delay between initial oncological consultation and the initiation of treatment between the FA-PCR group (16 days [11;18]) and the two SOP groups For the SOP group with unknown BRAF mutational status the delay was 26 days ([20;46], *p* = 0.035), for the SOP group with known BRAF mutational status it was 19 days ([4;33], *p* = 0.846).

For patients in the SOP group with unknown *BRAF* mutational status (11/29 patients, 38%), the delay between initial oncological consultation and initiation of treatment was 26 days [20;46], significantly longer than the delay observed for the FA-PCR group (*p* = 0.035, Figure 1).

For patients in the SOP group with known *BRAF* mutational status at initial consultation (18/29 patients, 62%), the delay in initiation of anti-*BRAF* therapy was 19 days [4;33], which was not significantly different from that observed for the FA-PCR group (*p* = 0.846, Figure 1).

## DISCUSSION

We demonstrate that in the vast majority of cases FA-PCR is able to determine *BRAF* mutational status within the same day as reception of the sample. This result is solely due to the analytical technique used and reflects the efficacy of this technique. We aimed to assess whether rapid determination of *BRAF* mutational status had an effect on the overall care of patients with metastatic melanoma. Since the time taken to commence treatment with BRAF inhibitors after determination of *BRAF* mutational status will vary depending on many different factors other than the analytical method used, the delay in initiation of treatment appeared to be the main outcome of this effectiveness and was retained as our primary objective. In this way, we demonstrated that the FA-PCR not only allowed decreasing the time of analytical technique with the delay between reception of FFPE sample and reception of the results by the oncologist but also speeding up the overall care of patients with the delay in initiation of treatment. The delay in initiation of treatment is reduced by 10 days, decrease partly explains by the reduction of 7 days for analytical technique.

For most of the samples, use of FA-PCR enabled us to determine *BRAF* mutational status within the same day, proof of the efficacy of this method. This rapid determination of *BRAF* mutational status led to a reduction in the time taken between initial consultation and the initiation of treatment, and the treatment delay for these patients was found to be consistent with that which we observed for patients with known *BRAF* mutational status at initial consultation. Therefore, using the FA-PCR platform to assess *BRAF* mutational status enabled us to commence personalized treatment with the same time delay as for patients with known *BRAF* mutational status at the time of consultation.

Use of FA-PCR clearly reduces the delay from sample to result compared to standard PCR, next generation sequencing (NGS) or IHC. Most laboratories analysing *BRAF* mutations by PCR or NGS process samples in batch, which implies that only one or two BRAF determinations are run per week. Therefore, mutation analysis using standard procedures is slower due to batch processing and increased hands-on time (DNA extraction, preparation of PCR mix, real-time PCR runs and data analysis). Immunohistochemistry is a fast assay for the determination of BRAF V600E expression, but it is limited to V600E detection, although a few cases of cross-reactions with V600K or V600D have previously been described [10]. The main weakness of our study is that there would have been an inherent bias to expedite the FA-PCR samples for analysis once the study team was aware of which samples were being processed, whereas there would have been less pressure to expedite samples from the retrospective SOP groups.

The number of patients in our study is limited in both groups. Despite this limited sample size, the population is representative since it is comprised of consecutive patients diagnosed with advanced or metastatic melanomas between January 2013 and November 2015 for retrospective group and between December 2015 and March 2016 for prospective one. A longer period would have increased the sample size and consequently the power. But, the number of subjects was sufficient to demonstrate statistically significant difference due to the large expected difference of delay between the two groups: for the delay between reception of the FFPE samples and receipt of the results by the oncologist, the difference between the two groups was highly significant (*p* < 0.001) with an a posteriori power of 98%.

To our knowledge, this study is one of the first to use FA-PCR for *BRAF* mutation screening with a view to improve the patient care pathway. Using FA-PCR on FFPE samples reduces the delay in treatment, but this delay can be highly increased for patients with no biopsy material available. For these patients, liquid biopsy may be a good alternative especially since BRAF ctDNA cartridges are available for Idylla™ FA-PCR system. However, concordance between liquid biopsy and FFPE samples should be prospectively investigated [11].

It is unlikely that progression-free survival and overall survival will be impacted by rapid diagnosis of *BRAF* status by FA-PCR. If treatment is implemented earlier, we can easily assume that patient care will be improved, but further investigation is needed to validate this initial finding with a larger number of patients and to assess the impact of rapid screening by FA-PCR on patient outcome, especially quality of life. Indeed, in our study, the two groups were not comparable in terms of prognosis, since most of the patients in the FA-PCR group had a stage IIIC disease at inclusion and most of the patients in the SOP group had a stage IV disease.

In conclusion, our study shows that using FA-PCR could improve the patient care pathway. Our data show that for patients with an available *BRAF* mutational status at the time of consultation or for those with an FFPE sample available at the time of consultation (thus enabling *BRAF* status to be determined by FA-PCR), treatment can commence earlier. The main aim of this study was to establish if an accelerated PCR assay was of interest for the management of patients with metastatic melanoma and it has been achieved. Further investigation is needed to determine whether plasma or fresh tissue samples can be used for the assessment of *BRAF* status by an FA-PCR platform if an FFPE sample is unavailable at the time of consultation.

## MATERIALS AND METHODS

This single center observational prospective study was conducted at the Institut de Cancérologie de Lorraine (France), from December 2015 to March 2016. The trial was approved by the institute’s scientific and ethical board. All methods were performed in accordance with the relevant guidelines and regulations. All data were de-identified prior to data analysis.

To be eligible for this study, patients had to be over 18 years of age, have a histologically proven advanced stage IIIC or metastatic stage IV skin or choroidal melanoma, and have a performance status score of 0 or 1 (Table 1). Patients with mucosal and uveal melanoma were not included.

Patients were included in this study from their initial oncological consultation after diagnosis of advanced or metastatic melanoma. Analysis of the mutational status of the *BRAF* gene was prescribed by the oncologist at this time. Analyses were performed either using the Idylla™ platform (Biocartis^®^, Mechelen, Belgium) on FFPE samples (FA-PCR group) or by using standard operating procedures (SOP group). For one patient, with no available sample, a biopsy was performed three days after the oncological consultation.

Tumour specimens were macrodissected after haematoxylin-eosin slide examination by a qualified pathologist to evaluate the percentage of tumour tissue in the sample prior to DNA extraction. For samples analysed by SOP 10 µM FFPE sections were used for DNA extraction, there was no restriction on the tumour tissue content of the samples from this group. For samples analysed by FA-PCR using the Idylla™ platform 10 µM FFPE sections were also used as samples (DNA extraction is automatically performed by the Idylla™ platform prior to PCR).

The Idylla™ platform (Biocartis, Mechelen, Belgium) is a cartridge-based fully automated platform that uses microfluidic processing with all reagents on-board. The platform is composed of a console and up to eight independent processing units allowing eight samples to be analysed at the same time (each sample can be screened for a different gene mutation e.g. *BRAF*, *KRAS*, *NRAS* or *EGFR* mutations in different samples).

For the Idylla™ BRAF assay, all melanoma samples were screened for the detection of p.V600E (c.1799T>A; p.Val600Glu), p.V600E2 (c.1799_1800delinsAA; p.Val600Glu), p.V600D (c.1799_1800delinsAT and c.1799_1800delinsAC; p.Val600Asp), p.V600K (c.1798_1799delinsAA; p.Val600Lys), p.V600R (c.1798_1799delinsAG; p.Val600Arg) and p.V600M (c.1798T>A; p. Val600Met) mutations. One section of macrodissected FFPE tumoral sample was spread between two wetted (nuclease-free water) filter papers, placed in the Idylla™ *BRAF* mutation test cartridge and then introduced into the instrument. According to Idylla™ manufacturer’s recommendations, the sample introduced into the cartridge must contain at least 50% tumour cells and have a surface area of 25–300 mm [2]. Multiple 10 µm sections and macrodissection were used for samples that did not meet these criteria, to ensure a total content of 50% tumour cells. All the different steps (DNA extraction, PCR and curve interpretation) were automatically performed by the Idylla™ system in less than 90 minutes [10]. Samples from the SOP group were analysed using the SYBRGreen assay as previously described [10]. Briefly, 20ng of tumoral DNA was extracted using the FFPE DNA extraction kit (Qiagen, Hilden, Germany) and *BRAF* mutations p.(Val600Glu), p.(Val600Lys) and p.(Val600Arg) were detected using specific probes.

Our primary objective was to assess the time taken (delay) from initial consultation to the implementation of treatment for patients with *BRAF* mutated advanced or metastatic melanoma, using FA-PCR to screen FFPE tissue samples for *BRAF* mutations (FA-PCR group).

Our secondary objective was to assess the time taken (delay) between reception of the sample and *BRAF* mutation results. We compared samples from a prospective group of patients analysed using the FA-PCR platform (FA-PCR group), to samples from a retrospective group analysed by SOP (SOP group). The retrospective group was comprised of consecutive patients diagnosed with advanced or metastatic melanomas between January 2013 and November 2015.

Results were expressed as median and inter-quartile range and were compared between groups according to the Mann-Whitney *U* test. Statistical analyses were performed using the SAS software version 9.4 (SAS Institute Inc., Cary, NC, USA.). The threshold for statistical significance was set to *p* < 0.05.
